# HCV-Induced Hepatocarcinogenesis: Molecular Mechanisms, Persistent Cancer Risk, and Future Perspectives

**DOI:** 10.3390/biomedicines14061295

**Published:** 2026-06-07

**Authors:** Snežana Jovanović-Ćupić, Milena Krajnović, Lidija Todorović, Ana Božović, Daniel Galun

**Affiliations:** 1 Laboratory for Radiobiology and Molecular Genetics, Vinča Institute of Nuclear Sciences, National Institute of the Republic of Serbia, University of Belgrade, Mike Petrovića Alasa 12-14, Vinča, 11351 Belgrade, Serbia; cupic@vin.bg.ac.rs (S.J.-Ć.); lidijat@vin.bg.ac.rs (L.T.); anabozovic@vin.bg.ac.rs (A.B.); 2 Clinic for Digestive Surgery, First Surgical Clinic, University Clinical Center of Serbia, 11000 Belgrade, Serbia; galun95@gmail.com; 3 School of Medicine, University of Belgrade, 11000 Belgrade, Serbia

**Keywords:** HCV, HCC, host immune response, epigenetics, post-SVR risk, biomarkers, therapy

## Abstract

Chronic infection with the hepatitis C virus (HCV) is the most significant risk factor for the development of hepatocellular carcinoma (HCC). It has been shown that the progression of HCV-related liver disease is mediated by both viral and host-specific factors. The HCV replication cycle is a host-dependent process that relies on intracellular signalling pathways within target cells. Thus, intracellular signal transduction plays a pivotal role in the modification of interactions between the host and HCV. These pathways are key regulators of liver diseases, including cirrhosis and HCC. In addition, HCV induces epigenetic modifications in the host genome that inhibit the expression of various tumour-suppressor genes. Some of these changes persist even after successful antiviral treatment and represent a continued risk for HCC development. Despite significant progress in the management of chronic HCV infection, this challenge remains unresolved. In this narrative review, we summarise the mechanisms of HCV-induced disease progression, focusing on the host immune response, the regulatory roles of viral and cellular proteins, and viral survival strategies during chronic infection. We also discuss HCV-induced epigenetic alterations that contribute to hepatocarcinogenesis both during infection and after viral clearance. These insights are important for identifying novel, reliable molecular biomarkers for patient surveillance and for designing new therapeutic approaches.

## 1. Introduction

The hepatitis C virus (HCV) is a positive-stranded RNA virus that primarily infects human hepatocytes and was first discovered in 1989 by Choo et al. [1]. It is estimated that 60 to 70 million people worldwide are affected [2]. Also, approximately 50–80% of cases with acute HCV infection progress towards a chronic state, with considerable risk of developing liver fibrosis, cirrhosis, and hepatocellular carcinoma (HCC). Early diagnosis is highly significant for the course and outcome of HCV infection. The introduction of direct-acting antivirals (DAAs) has considerably changed the treatment of chronic hepatitis C (CHC) over the last decade. Nevertheless, as of 2020, around 60 million people are still living with HCV infection [3,4]. Each year, about 1–4% of patients with cirrhotic CHC develop HCC, which is the most common type of primary liver cancer and has the third-highest cancer-related mortality rate [4].

Recent investigations suggest that in 2021, HCV-associated HCC caused around 150,000 deaths [2]. Furthermore, high genetic variability of HCV has resulted in eight distinct genotypes and numerous subtypes. Evidence indicates that treatment with DAAs effectively targets more prevalent strains (such as 1a, 1b, and 3a) but may be ineffective against non-epidemic subtypes. Unfortunately, the correct global and regional prevalence of uncommon HCV subtypes remains incomplete due to a lack of data across African and Asian nations [5,6]. These unusual subtypes exhibit genetic variations that make them less responsive or entirely resistant to standard antiviral drugs [6]. Several factors, including diagnostic barriers and the absence of a vaccine, keep HCV at the forefront of global health concerns. This is associated with the virus’s ability to evade immune surveillance, including viral mutation and inhibition of innate immune cells by HCV proteins. HCV, an obligate intracellular pathogen, highly depends on its host cells to propagate successfully. The HCV life cycle comprises several stages, including viral entry, protein translation, RNA replication, viral assembly, and release. Characterisation of numerous cellular factors involved in the HCV life cycle has provided extensive insight into HCV replication strategies. Some of these cellular factors are targets for anti-HCV therapies [7,8]. However, the interaction between HCV and host cells determines viral replication, progression of liver disease, and immune control. Moreover, HCV has been shown to induce extensive long-lasting epigenetic modifications in the host genome, thus silencing key tumour-suppressor genes [9,10]. Therefore, even with successful treatment, the risk of liver cancer persists for many patients [8]. Considering all these factors, HCC remains one of the major global health challenges. This type of cancer is characterised by high chemoresistance and late-stage diagnosis. In addition, its treatment is hindered by vast disparities across countries’ healthcare systems, including inconsistent screening and surveillance protocols, therapeutic options and state insurance policies. Hence, there is an urgent need to develop new follow-up and therapeutic approaches [11,12].

A growing number of studies indicate that the progression of HCV-related liver disease is mediated by both viral pathogenesis and host-specific factors (summarised in [9,10]). In this article, we focused on host–HCV interactions in CHC. We examined the influence of immune responses against HCV, which are regulated by both viral and cellular proteins. We also analysed the effects of modern antiviral DAA therapy on HCV proteins as well as mechanisms of viral escape from immune response, which lead to CHC infection and progression of liver fibrosis, cirrhosis, and HCC. In addition, we discussed the epigenetic pathways by which HCV promotes hepatocarcinogenesis during infection and after viral eradication, and outlined their implications for patient surveillance and the development of novel treatment approaches.

## 2. HCV–Host Interaction

### 2.1. HCV Life Cycle

The HCV RNA genome contains approximately 9600 nucleotides and one open reading frame (ORF), which is flanked by 5′ and 3′ untranslated regions (UTRs). Polyprotein is created when the viral RNA is translated. Different cellular and viral proteases break down this polyprotein into structural proteins, core (C) and envelope (E) E1/E2, and seven non-structural (NS) proteins: viroporin p7, NS2, NS3, NS4A, NS4B, NS5A, and NS5B [7,13].

It has been shown that HCV can infect hepatocytes through two distinct routes, including cell-free virus entry and direct cell-to-cell transmission. In addition, viral entry requires interactions among the components of lipo-viro particles (LVPs), particularly apolipoprotein E (Apo-E) and envelope proteins. A lipid envelope, E1 and E2 glycoproteins, core protein, and viral RNA combine to form a nucleocapsid. This complex contributes to viral entry. In chronic infection, the HCV particles circulate as LVPs. In fact, virions are linked to lipoproteins that contain apolipoproteins B (Apo-B) and Apo-E, such as low-to very low-density lipoproteins (LDLs and VLDLs) [14,15]. Moreover, these LVPs are biologically significant in altering lipid metabolic pathways to enable their assembly, circulation, and entry into host cells. Thus, the presence of apo-B and apo-E facilitates entry into hepatocytes via lipoprotein receptors [16,17]. Concerning immune evasion, HCV and LDL/VLDL particles can mask virus epitopes, making LVPs less visible to neutralising antibodies. Moreover, prolonged persistence of HCV in the host can lead to oxidative stress, DNA damage, and malignant transformation [18].

LVP formation can be prevented by targeting host factors such as Apo-E, Apo-B, and microsomal triglyceride transfer protein (MTP). According to earlier results, the LDL receptor, scavenger receptor class B type I (SR-BI), CD81, and claudin-1 (CLDN1) are exploited by LVPs. Thus, virus entry can be disrupted by medications or antibodies. LVPs can modulate apoptosis and the transport of anticancer agents. In experimental models, anti-SR-BI antibodies have the most effect. It has been reported in animal models that viral- and non-viral-based viral-like particles (VLPs) have potential for cancer treatment in preclinical studies [19]. Furthermore, statins and MTP inhibitors may attenuate HCV infectivity. Thus, statins may increase the efficacy of antiviral treatments. Research concerning vaccine development and monoclonal antibodies designed to recognise lipid epitopes remains a big challenge. Understanding the different mechanisms and biology of LVPs will help to purify vaccine design [19,20]. The first step of viral entry is characterised by low-affinity binding to two host receptors on the surface of a hepatocyte: low-density lipoprotein receptor (LDLr) and heparin sulfate proteoglycans (HSPGs). In this route, interactions occur between the external E1/E2 heterodimer membrane protein of HCV and SR-BI, tetraspanin protein CD81, and members of the CLDN family (1, 6, and 9) [7,21,22]. These processes initiate endocytosis. The virus envelope interacts with endosomal membranes, allowing the HCV genome to be released into the cytoplasm. Some human cell lines demonstrate that more cellular factors participate in viral entry [23]. Host factors, which protect cells from infection as EW1-2wint, play a significant role in HCV entry [23,24]. In addition, EW1-2wint stopped interactions between HCV glycoproteins and CD81 or blocked signalling pathways for HCV entry [21]. Studies on hepatocarcinoma cells that express LDLr, CD81, and SR-BI show maximal infectivity [22,25]. Likewise, previous studies demonstrate that HCV E1 protein and HCV infection promote pro-metastatic activity in cancer cells and participate in transcriptional downregulation and protein degradation of Nm23-H1. The Nm23-H1 as a host cell protein has been studied in a diversity of human cancers. This protein was discovered in a murine tumour model as the first metastasis suppressor gene [26].

The HCV envelope E1/E2 glycoproteins play a leading role in viral entry and represent the primary antigenic targets for neutralising antibody responses. In vitro expressed HCV E1/E2 heterodimers have shown incomplete structural and functional characterisation, which has limited their effectiveness as vaccine targets [27,28]. Despite these limitations, electron microscopy confirmed that the structure of the E1/E2 heterodimer in complex has three elementary neutralising antibodies. This complex could be an attractive target for vaccine and antiviral treatment development [29].

### 2.2. Translation of HCV

Regulation of gene expression is one of the most important steps in the viral life cycle. Thus, HCV polyprotein translation starts when ribosomal subunits attach to HCV RNA within the endoplasmic reticulum (ER). The ribosome–RNA complex then anchors to the ER membrane, where translation is completed. This process produces a single polyprotein of about 3000 amino acids in length [30,31]. The translation process of the HCV genome is controlled by three main factors: the physical shape of the viral RNA itself, the cell’s standard protein-building hardware (40S ribosomal subunit and initiation factors), and specific cellular regulators such as micro-RNA (miR)-122 and helper proteins known as internal ribosome entry site (IRES) trans-acting factors (ITAFs) [32].

It has been reported that the HCV genome lacks a 5′ cap structure. Instead, HCV translation is regulated by IRES, which is located in the 5′ UTR, part of the core protein coding sequence, and by the 3′ UTR [30,32]. Thus, with the help of IRES, the genome ends are open to translation, and HCV replication signals can be positioned at the termini [7,32,33]. In addition, the liver-specific miR 122 stimulates HCV IRES-dependent translation and stabilises a certain structure of IRES [32,34].

### 2.3. Targeting HCV RNA Structures with Small Molecules

It has been reported that HCV RNA plays a crucial role in viral life cycles, particularly in translation, replication, and gene expression regulation. Also, RNA-targeting therapeutics are an attractive mode for expanding target space in the human genome. Additionally, small molecules have the potential for oral distribution and permeation across the blood barrier. The advantage is that the small molecules are more susceptible to drugs than nucleic acids and have better physicochemical characteristics [35]. These molecules interact with different RNA substrates, RNA structural motifs, RNA enzymes, or specific RNA sequences. Furthermore, the new DAA therapy can stop HCV translation and replication by directly blocking viral proteins or by targeting the host factors, such as miR-122 antagonists (miravirsen) and IRES-targeting small molecules [36,37].

### 2.4. HCV Replication, Assembly, Release, and Therapy Inhibitors of HCV Replication

**Figure 1:**
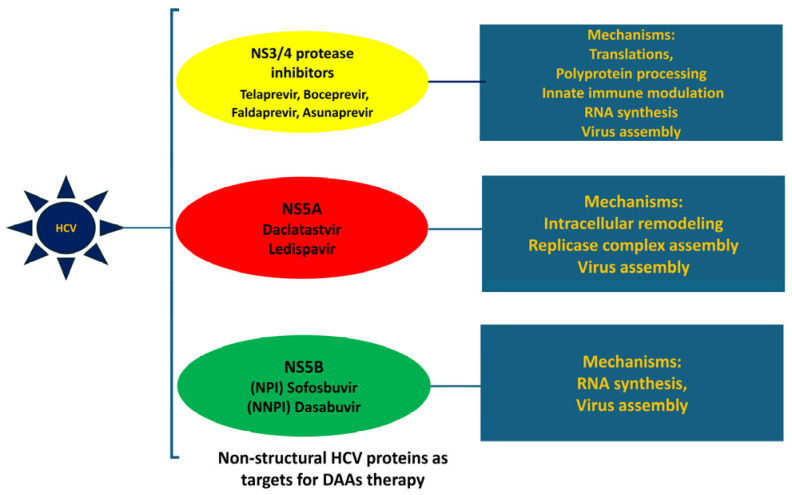
Target sites of direct-acting antivirals (DAAs) within the hepatitis C virus genome. HCV: hepatitis C virus, NS: non-structural protein, NPI: nucleotide polymerase inhibitor, NNI: non-nucleoside inhibitor.

The development of robust cell culture models for HCV provides an examination of the structure and functions of viral replication sites in depth (reviewed in [33]). Early pioneering electron microscopy studies of liver tissue from infected patients and chimpanzees suggested that HCV, like other positive-strand RNA viruses, induced membrane modifications in infected hepatocytes [33]. Like other positive-polarity single-stranded viruses, HCV causes membrane modification, which is known as a membranous web [23,38]. It was shown that expression of NS proteins, especially NS4B, results in vesicle accumulation, thereby creating a membranous network [39]. These findings suggest that all virus proteins assemble on the membranous web and form a replication complex in HCV-infected cells. Furthermore, synthesis of a negative-strand RNA with NS5B and RNA-dependent RNA polymerase (RdRp) is a critical step in virus replication. The replication complex consists of a membranous web composed of host cell proteins and NS. This complex is in contact with perinuclear membranes and thus takes part in replication and post-translational processing [23]. The main roles of HCV proteins in replication are summarised in the review by Li et al. [7]. In recent years, different host cell factors have been discovered (reviewed in [40]). These host cell factors (proteins, lipids, and nucleic acids) participate in the replication of HCV. The newly formed virions are released in the pericellular space via exocytosis, while in the Golgi apparatus, nucleocapsids are covered and matured. Lastly, the HCV genome encapsulation occurs in the ER [7,41]. Current antiviral therapy acts on the life cycle of HCV and can be classified into three main categories. NS3/4A protease inhibitors stop the cleaving of the HCV polyprotein in the ER and block the formation of mature viral proteins. NS5A inhibitors induce accumulation of these proteins in the ER, and they stop virus replication and assembly. NS5B inhibitors target the RdRp and inhibit the translation and replication of the virus. Finally, a nucleotide analogue can be incorporated into the RNA strand and block synthesis at the same time [8] (Figure 1).

### 2.5. Host Immune Response to HCV Infection

The disease progresses due to the interaction between the virus and the host’s immune system. The human system has developed innate and adaptive immune responses to protect against HCV infection. The first step of the innate immune response is virus recognition. The host’s immune system identifies some structural and NS HCV proteins through receptor retinoic acid-inducible gene-I (RIG-I)-like receptors and toll-like receptors (TLRs), which recognise viral pathogen-associated molecular patterns and trigger the production of antiviral cytokines such as interferons IFNs [42]. Thus, key components include IFNs (types I and III), IFN-stimulated genes (ISGs), and adaptive immune cells. These IFNs act by activating signal transducers and activators of transcription (STAT)-1 and STAT-2, which combine to form the ISGF3 factor. This complex then moves to the nucleus and begins with the synthesis of IFN-stimulated antiviral genes. These genes inhibit HCV replication and activate natural killer (NK) cells. It has been reported that high pretreatment ISG levels are associated with poor response to therapy [43]. Resistance to ISGs increases neutralising antibody responses, thereby enabling HCV variants to escape selection pressures [44,45]. The NK cells may produce antiviral cytokines or be cytotoxic at higher levels of killer immunoglobulin-like receptors (KIRs) and perforin [42,46]. The NK cells represent the connection between innate and adaptive immunity. There is evidence that in the progression of liver inflammation, dendritic cells (DCs) and Kupffer cells participate. In the innate immune response, plasmacytoid DCs and myeloid DCs produce higher levels of type I IFNs (IFNA and IFNL) and activate the adaptive immune response [47]. During infection, HCV proteins, specifically core and p7, stimulate the release of IFN1B from Kupffer cells acting as human macrophages [48,49]. The bond between innate and adaptive immune responses to HCV infection is T cells.

In control of infection are specific CD8+ T cells and cytotoxic T lymphocytes (CTLs). The first response to acute HCV infection begins when naïve T cells are filled with HCV antigens. These cells undergo expansion and contribute to the clearance of infection by specifically killing infected cells [50,51]. In addition, small subsets of HCV-specific CD8+ T cells can persist as long-lived memory cells. These cells can be recalled upon re-exposure to the same pathogen. Also, during virus replication, the cytokines secreted by T cells have effects on the outcome of infection. Furthermore, previous studies suggest that HCV-specific-CD4+ T cell responses are associated with spontaneously resolved HCV infection [42,51]. Depending on the course of the HCV infection, some authors suggest that peripheral CD4+T cells have two different effector phenotypes. One phenotype is characteristic in patients who can spontaneously resolve HCV infection, while in patients with persistent infection, other types of phenotypes dominate [42,51,52].

The persistent infection leads to weakening or absence of the T cells. This multistep process depends on responses to virus peptides and mitogens [51,53]. Some studies have shown that during CHC infection, T cells frequently enter a state of exhaustion, resulting in reduced cytokine production and impaired cell division (reviewed in [51]). Accumulations of exhausted T cells with inhibitory receptors may explain why the immune response often fails in patients with CHC infection [42,51]. Additionally, during HCV infection, T-helper 1 (Th1) cells trigger the differentiation of B cells into plasma cells and produce neutralising antibodies. These antibodies are often insufficient to control the infection, especially due to the high variability of the virus. It is assumed that early treatment with DAA therapy can partially prevent immune exhaustion and result in intense HCV-specific responses after treatment. Furthermore, patients with sPpontaneous clearance of infection had wide HCV-specific responses [54]. Moreover, early treatment with DAA therapy can repair the immune system and prevent the occurrence of HCC. Likewise, patients with sustained virological response (SVR) had a lower risk of developing HCC than non-responders (NRs) [42,51]. Understanding the interactions between innate and adaptive immune responses may be significant for the development of an efficient prophylactic vaccine, which would stimulate protective immunity and limit transmission of infection [42,45].

Virus-escape mutations make HCV unique. Consequently, about 50% to 70% of people with CHC have mutations in the virus at certain CD8+ T cell epitopes. These changes occur during the initial phases of infection and persist within the virus quasispecies over the long term, acting as a primary carrier for immune evasion and the development of chronic disease [55,56].

### 2.6. New DAA Therapies for HCV Infection and the Resistant HCV Variants

The introduction of DAAs has significantly improved HCV treatment. These drugs are more effective than older IFN-based therapies. The first-generation DAAs, protease inhibitors boceprevir and telaprevir, were designed to block viral replication. They significantly improved treatment for patients with CHC [57]. Nevertheless, this therapy also has limitations, including the risk of viral resistance, adverse effects, and complex treatment regimens. Mutations in the NS3/4A protease and resistance-associated variants often emerged during treatment, making the drugs less effective [58]. In addition, boceprevir and telaprevir were not administered as standalone therapies. In most patients, they were combined with pegylated interferon and ribavirin (PEG-IFN/RBV) and ribavirin, which brought additional adverse effects and prolonged treatment duration [59,60].

The second and third generations of DAA treatment are very successful, with 95–100% of patients achieving SVR. Thus, combinations of ledipasvir–sofosbuvir have milder side effects than older IFN-based treatments. These drugs are effective against genotypes 1 and 3, and these patients already have a chance to achieve SVR [57]. Moreover, DAA therapies are highly effective in patients with cirrhosis and liver transplants and those who have relapsed after previous treatment [61]. The latest generation of HCV therapy is pan-genotypic DAAs. The new pan-genotypic DAAs are designed to target multiple genotypes. These therapies have taken an important step forward in simplifying treatment for all major HCV genotypes. Thus, the genotype testing before initiating therapy is reduced. Based on the above facts, patients with pan-genotypic therapy achieve high SVR rates, better tolerability, simplified treatment regimen, and shorter treatment durations (8 or 12 weeks) (reviewed in [57]). Some authors found that polymorphisms in NS5A and NS3 regions, which affect pre-existing resistance-associated substitutions (RASs), increase the risk of resistance to standard pan-genotypic DAA treatments [6,62]. Also, in vitro data suggest that some of the uncommon HCV subtypes are susceptible to certain drugs (e.g., elbasvir and velpatasvir), while 3b, 3g, 1l, and 6u had lower sensitivity to standard pan-genotypic inhibitors [6]. In addition, genotype 3b and 3g subtypes were inherently more resistant to NS5A inhibitors [63,64]. Kattel et al. [65] noticed that in patients with genotype 3, the baseline frequency of RASs was 37% in the NS3, 29% in the NS5A, and 15–24% in the NS5B region of HCV genomes. It has been reported that subtypes like 4r with baseline RASs (L28V, L30R, and M31L) confer resistance to several NS5A inhibitors [66]. The standard pan-genotypic therapy is highly effective against subtype 6a, with cure rates above 95% to 98%, but several rare strains possess inherited resistance to standard DAA therapy. These polymorphisms impair antiviral drug binding and thereby reduce treatment efficacy [6]. Data on the resistance profiles of uncommon genotypes are limited and are based on small patient cohorts from Western countries [6,67].

Despite advances in new therapy, the early detection, prevention and treatment of HCC remain significant clinical challenges. Recent research suggests that HCV infection induces metabolic alterations, lipid dysregulation, and mitochondrial dysfunctions in the development of HCC [68]. Furthermore, complete control of HCV infection cannot be achieved with current DAAs. That is why, in adequate fighting to eliminate HCV, we need to focus on HCV vaccine development [3,68].

## 3. Epigenetic Changes in HCV-Induced HCC

**Figure 2:**
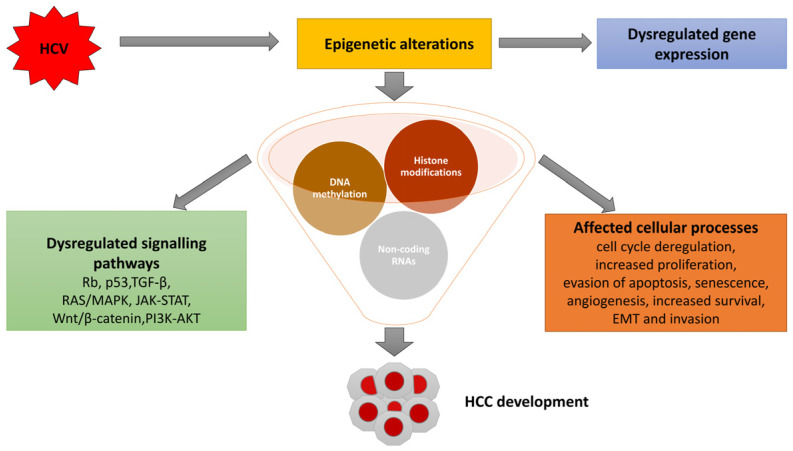
Epigenetic mechanisms in HCV-related hepatocarcinogenesis. HCV induces epigenetic modifications in the host genome, causing the dysregulation of key signalling pathways and critical cellular processes, which leads to tumour development. HCV: Hepatitis C virus, HCC: Hepatocellular carcinoma, EMT: Epithelial-to-mesenchymal transition.

Beyond disrupting the host immune system, metabolism, and genome stability, HCV induces profound epigenetic modifications in the host genome, creating a pro-carcinogenic environment and contributing to the long-term risk of HCC [9,10]. Epigenetics refers to heritable changes in gene expression that occur without altering the DNA sequence. These mechanisms include genomic DNA methylation, post-translational histone modifications, and regulation of gene expression by non-coding RNAs (ncRNAs) [69,70]. By switching genes on or off, epigenetic alterations fundamentally dysregulate host cellular processes critical to both the HCV life cycle and hepatocarcinogenesis [71] (Figure 2).

Growing evidence indicates that epigenetic alterations emerge early during CHC infection and become more frequent as the disease progresses through fibrosis and cirrhosis towards HCC [72,73,74]. Moreover, these modifications can leave a persistent epigenetic signature in the host genome even after viral clearance, and contribute to a sustained risk of developing HCC despite successful antiviral therapy [71,75,76,77]. Considering that, monitoring HCV-induced epigenetic alterations in the host genome enables the tracking of disease progression [74]. Therefore, elucidating the underlying molecular mechanisms of hepatocarcinogenesis is essential for improving clinical treatments and discovering new, sensitive diagnostic biomarkers.

### 3.1. DNA Methylation in HCV-Induced HCC

DNA methylation is an essential epigenetic modification and a primary regulator of gene expression. This process involves the addition of a methyl group to the fifth carbon of cytosine within the promoter CpG islands, and is catalysed by DNA methyltransferases (DNMTs). In this way, DNA methylation silences crucial tumour-suppressor genes in various types of human cancer, including HCC [78].

Research into the mechanisms of HCV-related hepatocarcinogenesis has shown that key signalling pathways in HCC, such as Rb, p53, TGF-β, RAS/MAPK, Wnt/β-catenin, JAK-STAT, and PI3K-AKT, are often compromised by DNA methylation-induced silencing of the involved tumour-suppressor genes [77,79,80,81,82,83,84,85,86]. Their inactivation disrupts fundamental cellular processes normally regulated by these pathways [87].

There is evidence that HCV viral proteins, including core, NS3, and NS5a, can epigenetically inhibit the expression of various tumour-suppressor genes [79,84,88,89,90,91,92,93,94], with the role of the core protein being the best studied to date. In particular, HCV core protein upregulates DNMT expression through two primary mechanisms: (1) induction of the activator protein 1 (AP-1) complex through MAPK signalling [84], and (2) activation of the Rbpathway, which stimulates DNMT1 expression [79]. Studies have identified HCV genotype-specific variations in both mRNA and protein levels of DNMT1 and DNMT3 [95,96]. Furthermore, evidence suggests that the HCV core protein inhibits gene transcription through different epigenetic mechanisms. For instance, it represses *p16* expression by increasing DNMT1 and DNMT3b levels [84,94], but triggers *RASSF1A* methylation by inducing the histone methyltransferase SET and MYND domain containing 3 (SMYD3) [94,97].

Studies on experimental in vitro and in vivo models have established that HCV epigenetically inhibits genes involved in cell cycle control, epithelial-to-mesenchymal transition (EMT), and apoptosis. Research demonstrates that HCV core protein induces promoter hypermethylation of *p16*, *GADD45β*, and *p14* genes through upregulation of DNMTs, leading to impaired cell cycle regulation, DNA repair, and apoptosis, respectively [82,83,84,86].

In addition, HCV triggers EMT by suppressing the *SFRP1* and *CDH1* genes through the involvement of DNMTs, histone deacetylase 1 (HDAC1), and methyl-CpG-binding domain (MBD) proteins, which facilitate cell migration and contribute to HCC progression [79,85,98]. Importantly, it has been shown that these oncogenic effects can be reversed using epigenetic inhibitors, such as the DNMT inhibitor 5-azacytidine and the histone deacetylase sirtuin 1 (SIRT1) inhibitor sirtinol, or by DNMT knockdown, highlighting the therapeutic potential of targeting these pathways [79,82,85,98].

So far, extensive research on clinical samples, including single-gene analyses and genome-wide methylation profiling, has provided important insights into HCV-related liver cancer. Many studies have shown that this process is driven by aberrant hypermethylation of multiple genes, suggesting a specific methylation profile associated with HCV. These patterns can serve as important markers for HCV-related HCC, despite some inconsistencies in the literature, likely due to differences in HCV genotype, geographical variation among study groups, and the sensitivity of detection methods [74,99,100].

In an early study, Yang et al. [100] linked the methylation of *p15*, *SOCS-1*, and *APC* specifically to HCV-induced HCC. Zhang et al. [101] reported that such alterations in *p15*, *p16*, and *RASSF1A* are detectable in the serum of HCV-infected patients up to nine years before clinical HCC diagnosis, proposing them as promising biomarkers for early detection. Subsequently, Iyer et al. [102] detected high methylation frequencies in the *p15*, *p16*, *FHIT*, and *CDH1* genes in both tissue and plasma from HCC patients, establishing plasma-derived DNA as a reliable, non-invasive surrogate source for methylation profiling in HCC.

Zekri et al. [74] demonstrated that promoter methylation of *p14*, *p15*, *p73*, and *MGMT* genes detected in plasma correlates with the progression of HCV genotype 4-related liver disease, proposing these alterations as candidate biomarkers for monitoring the transition from CHC to cirrhosis and HCC. Their expanded study on HCV genotype 4 patients further revealed that methylation frequencies of multiple genes, including *p14*, *p73*, *RASSF1A*, *CDH1*, and *MGMT*, significantly increase as the disease advances, while *APC* methylation was more prevalent in CHC cases. Given the high concordance in observed methylation frequencies between tissue and peripheral blood lymphocytes (PBLs), the authors proposed a diagnostic panel comprising *APC*, *p73*, *p14*, and *MGMT* as a highly sensitive and specific tool for distinguishing HCC from CHC.

A recent meta-analysis by Zhang et al. [103] indicated a stage-dependent epigenetic progression driven by HCV infection. Specifically, *p16* methylation was observed as an early-stage event, present in both malignant and adjacent non-tumourous tissues, whereas hypermethylation of the *GSTP1*, *APC*, and *RUNX3* genes was restricted to tumour tissues, suggesting their critical role in the later stages of carcinogenesis.

Beyond these panels, *RASSF1A* gene is an important marker because of its exceptionally high methylation frequencies in both HCC and CHC, highlighting its pivotal involvement in HCV-related hepatocarcinogenesis [74,104]. Multiple studies have shown that *RASSF1A* methylation levels consistently increase with disease progression from CHC to cirrhosis and HCC, and can be detected in tissue and blood across the various stages of liver disease, reinforcing its potential for early monitoring and detection in persons at risk of HCC [74,104,105,106,107].

In addition to these findings, extensive research in recent years has identified further tumour-suppressor genes commonly methylated in HCV-induced HCC, including *RASAL1*, *EGLN3*, *CSMD1*, *BCORL1*, *SFRP1*, *ZNF382*, *RUNX3*, *LOX*, and *RB1*. Suppression of these genes directly contributes to hepatocarcinogenesis, as recently reviewed [108].

Genome-wide methylation studies have provided broader insight into HCV-induced global host DNA methylation patterns. In an earlier study, Deng et al. [109] identified abnormal methylation of tumour-suppressor genes in the RAS/MAPK and Wnt/β-catenin signalling pathways, suggesting a potential prognostic role in HCV-driven hepatocarcinogenesis. Subsequently, Shen et al. [110] detected more than 130,000 CpG sites with significantly altered methylation in tumours compared with adjacent normal tissue, proposing that a subset of 500 CpG sites could serve as a precise diagnostic tool for identifying malignant tissue. Okamoto et al. [111] demonstrated that HCV infection induces significant, genome-wide DNA methylation changes in mouse models with humanised liver, with the extent of these alterations increasing with the duration of infection. In addition, Wijetunga et al. [112] found that HCV infection induces methylation at hepatocyte-specific enhancer regions, which, together with polycomb-mediated repression, leads to decreased expression of target genes at specific genomic sites. These epigenetic changes occur early during HCV infection and establish a pro-carcinogenic liver field effect, suggesting that targeting epigenetic regulators could be a promising strategy for cancer prevention. Recent research by Goncharova et al. [113] showed that DNA methylation patterns in HCV-associated HCC differ depending on the underlying liver condition. Their study indicated that HCCs originating from cirrhosis exhibit more complex methylation changes than those arising from fibrosis or non-viral aetiologies. Specifically, while cancer driver genes such as *APC*, *p15*, *GSTP1*, *ELF4*, *TERT*, and *WT1* showed hypermethylation only in the presence of cirrhosis, other genes such as *ZNF154* and *ZNF540* remained consistently hypermethylated regardless of whether the tumour originated from fibrotic or cirrhotic tissue.

Management of CHC and its complications depends on successful antiviral therapy and early detection of reliable molecular markers in at-risk individuals [114,115]. Previous research suggests that HCV-induced epigenetic alterations in the host genome, particularly changes in DNA methylation patterns, can significantly influence the response to IFN-based therapy [99]. For instance, Zekri et al. [99] identified *MGMT* methylation as a predictor of non-response to combined PEG-IFN/RBV therapy in patients with HCV genotype 4, while Mostafa et al. [116] further observed that hypermethylation of the *p16*, *RASSF1A*, *MGMT*, *SFRP1*, and *APC* genes significantly affects the response to the same regimen.

It has previously been shown that host genetic factors, such as polymorphism in the *IL28B* gene and variability of viral proteins within the same HCV genotype, influence the success of IFN-based antiviral therapy and disease course [117,118,119,120]. However, studies integrating these factors with the methylation status of specific tumour-suppressor genes are lacking. Evidence suggests that core amino acid substitutions at position 70 can predict treatment outcomes and serve as pretreatment predictors of HCC after DAA therapy [121,122,123]. Recently, our research group demonstrated that combined analysis of *RASSF1A* gene methylation, HCV core protein variability, and the *IL28B* rs12979860 polymorphism can predict treatment outcomes with PEG-IFN/RBV in patients with genotype 1b CHC [124]. The predictive value of these markers in the context of DAA therapy remains unknown. Although DAA therapy has significantly improved SVR rates compared with IFN-based regimens, it would be worthwhile to determine whether these parameters can indicate the risk of HCC development in both SVR and NR patients, irrespective of therapy.

### 3.2. Histone Modifications in HCV-Induced HCC

While DNA methylation is a relatively stable epigenetic change, histone modifications are more dynamic. Histone modifications are epigenetic posttranslational modifications that regulate gene transcription by modulating chromatin structure. In healthy cells, these modifications determine chromatin configuration, thereby enabling or blocking the recruitment of transcription factors. In contrast, HCV-induced histone modifications remodel chromatin configuration, leading to the activation of oncogenes and the silencing of tumour suppressors. Consequently, histone modifications persist in liver cells even after DAA treatment, and many patients remain at high risk of developing HCC long after the virus is eradicated. The most significant HCV-induced histone modifications are phosphorylation, acetylation, and methylation [89].

HCV triggers the aberrant recruitment of histone acetyltransferases (HATs), which catalyse acetylation of histone tails, thereby neutralising the positive charge of lysine residues on histones, consequently relaxing chromatin. The chromatin then becomes accessible to transcription factors that activate genes involved in proliferation and cell growth. For example, the HCV core protein directly binds the CBP/p300 protein, a histone acetyltransferase. Researchers propose a mechanism in which the Core-CBP/p300 complex indirectly activates the nuclear factor of activated T-cells (NF-AT1) and recruits other transcription factors, thereby activating genes related to cell proliferation, inflammation, and, eventually, HCC [125]. p300 catalyses histone H3K27 and H3K9 acetylation, leading to enrichment of H3K27ac (histone 3 lysine 27 acetylation) and H3K9ac (histone 3 lysine 9 acetylation) at gene promoters [126]. Consistently, HCV infection promotes H3K27ac enrichment, a marker of open chromatin, at gene promoters, and influences their overexpression [75,76,127]. Among them is the *SPHK1* promoter. SPHK1 catalyses sphingosine phosphorylation, forming sphingosine-1-phosphate (S1P), a bioactive lipid which contributes to anti-apoptosis and angiogenesis [75]. On the contrary, the loss of H3K27ac at the *TLR3* promoter (a sensor of HCV infection and a member of the IFN pathway) leads to *TLR3* gene silencing. Without this sensor, HCV-infected cells are not driven to apoptosis and the immune response is suppressed [127]. Furthermore, HCV-induced enrichment of H3K9ac, a marker of open chromatin, at regulatory elements (CpG islands and enhancers) leads to activation of genes involved in proliferation, angiogenesis, and inflammation [71,128]. On the other hand, HCV infection led to depletion of H3K9ac on the base excision repair pathway genes [128]. These histone remodelling processes persist even after DAA treatment and viral clearance, leaving epigenetic scars on human liver cells and increasing the risk of tumourigenesis and HCC [75].

HCV induces the enrichment of H3K4me1 (histone 3 lysine 4 monomethylation) at enhancers and H3K4me3 (histone 3 lysine 4 trimethylation) at promoters, which are markers of open chromatin. On the other hand, H3K27me3 (histone 3 lysine 27 trimethylation) and H3K9me (histone 3 lysine 9 monomethylation), markers of closed chromatin, are reduced in the HCV-infected cells [71,127]. However, Perez and colleagues [71] demonstrated that H3K4me3 was enriched in the gene body, e.g., exons and introns. H3K4me3 enrichment induces persistent activation of genes involved in proliferation, growth, angiogenesis, and immune response. Hlady et al. [127] demonstrated that HCV infection activates the same gene regions as IFN-α treatment and consequently leads to epigenetic scarring. HCV-induced H3K27ac enrichment and reduction in H3K27me3 genome-wide persisted even after the interferon-α signal was omitted and the virus was cleared. However, H3K27me3 gain throughout the *TLR3* gene locus and loss of H3K27ac at the same promoter lead to *TLR3* silencing, anti-apoptosis and immune response suppression, as noted previously. These findings were validated in liver biopsies from post-SVR HCV patients, confirming that the same IFNα-activated regions were remodelled after HCV infection and DAA treatment. That confirms that HCV leaves persistent epigenetic scars that remain after DAA treatment. Furthermore, the loss of H3K9me3 at promoters and TSSs, a marker of closed chromatin, reduces the expression of base excision repair proteins [128].

HCV core protein has also been shown to decrease monoubiquitination of H2AK119 via degradation by ring finger protein 2 (RNF2). RNF2 is a catalytic unit of polycomb repressive complex 1 (PRC1), which catalyses the ligation of ubiquitin to H2A, condensing chromatin and repressing gene transcription. When this break is removed by HCV, *HOX* gene promoters become accessible, and *HOX* gene transcription is activated. Overactivation of *HOX* genes drives cell proliferation, angiogenesis and inflammation and eventually leads to HCC. Treatment of HCV-infected cells with LY-411575, an indirect inhibitor of HCV core protein, restored H2AK119 and RNF2 and suppressed *HOX* genes expression [129].

HCV infection triggers histone modifications that block the cell repair mechanism. HCV upregulates protein phosphatase 2A catalytic subunit (PP2Ac). Increased levels of PP2Ac inhibit protein arginine methyltransferase 1 (PRMT1), thereby reducing H4R3me2 (histone 4 arginine 3 dimethylation). H4R3 methylation is required to tag chromatin. Without it, the cell cannot phosphorylate H2A histone family member (XH2AX) at the damage site and form γ-H2AX, which is a signal for DNA repair machinery. Consequently, the repair proteins could not recognise and bind to DNA damage sites, leading to double-strand breaks. The accumulation of DNA damage will eventually lead to genomic instability and, consequently, to HCC. Since the S-adenosyl-L-methionine (SAMe) partially reversed these changes in cell lines, the authors suggest it as a potential drug candidate for HCC [130]. All mentioned HCV-induced histone modifications are presented in Table 1.

### 3.3. Non-Coding RNAs in HCC-Induced HCC

NcRNAs represent an abundant and diverse group of RNA transcripts that do not encode proteins. Instead, they perform a wide range of regulatory functions in fundamental biological processes such as growth, development, and organ function, and also play a critical role in pathological changes and various diseases, including cancer [131]. NcRNAs function within complex gene regulatory networks, modulating gene expression at transcriptional, post-transcriptional, translational, and post-translational levels through interactions with each other as well as with DNA, mRNA, and proteins [132,133]. While the diversity of ncRNA species continues to expand, the most extensively studied groups remain miRNAs and long noncoding RNAs (lncRNAs). A group of abundant yet less-studied lncRNAs are circular RNAs (circRNAs). They are being increasingly investigated, particularly in the context of cancer.

MiRNAs (miRs), short ncRNAs of ∼22 nt in length, regulate gene expression at the post-transcriptional level. They bind to target mRNAs and induce their degradation or inhibit their translation into proteins. MiRs often regulate more than one target mRNA, and individual mRNAs are frequently targeted by several miRNAs. As such, miRNAs function as master regulators that control the expression of a broad range of coding and non-coding genes [131].

LncRNAs are non-coding transcripts longer than 200 nt, transcribed by RNA Pol II from independent promoters. Their structural complexity enables diverse interactions with DNA, RNA, and proteins. Different lncRNAs regulate gene expression at epigenetic, transcriptional, and post-transcriptional levels. Some of them function in the nucleus, acting as guides for chromatin-modifying complexes or transcription factors. Other lncRNAs are located in the cytoplasm, where they regulate protein levels, either by directly controlling mRNA stability or by acting as competing endogenous RNAs [131,134].

CircRNAs are characterised by a covalently closed loop structure, which lacks 5′ and 3′ ends. Due to circular configuration, they are highly stable and resistant to exonuclease-mediated degradation. Despite widespread expression of circRNAs, their roles and mechanisms of action are largely unknown. Evidence suggests that they can function as miRNA sponges, thereby preventing them from inhibiting their target mRNAs. Additionally, circRNAs can interact with RNA-binding proteins and affect their activity, localisation, or stability [131].

Multiple studies have shown that HCV infection dysregulates ncRNAs in host cells. HCV-induced ncRNA dysregulation arises from a combination of direct viral RNA interactions, epigenetic and transcriptional reprogramming, disruption of RNA processing, and chronic inflammatory signalling. Collectively, these alterations not only support viral replication but also are believed to contribute to the establishment of a pro-oncogenic cellular environment that promotes HCC initiation and progression [70,108,135]. A well documented example is miR-122, a liver-specific miRNA that plays a dual role in both facilitating HCV replication and modulating pathways of tumour suppression [136,137,138].

Accumulating evidence supports a significant role for ncRNAs in HCV-related hepatocarcinogenesis [134,139,140]. Over the past decades, hundreds of dysregulated ncRNAs have been identified in tissue and plasma samples from HCC patients. Some of them are well established oncogenic miRs, such as miR-21-3p and miR-92a-3p, which are frequently upregulated in other cancer types [141,142]. Others exhibit liver-specific expression patterns, such as miR-122, a tumour-suppressor miR that is often downregulated in HCC [143,144,145].

Many studies have further described the roles of specific ncRNAs in HCC development and/or progression, as reviewed elsewhere [140,146,147,148,149]. More recently, it was found that miR-21a-3p promotes migration and invasion of HCC cells and upregulation of YAP1 expression via direct inhibition of SMAD7, the negative regulator of the TGF-β signalling pathway [150]. Another study demonstrated that miR-92a-3p plays a tumour-promoting role in HCC by mediating the PI3K/AKT signalling pathway [151]. In a mechanistic study involving in vitro and in vivo models, as well as human HCC tissue samples, Cao et al. [152] have found that miR-19a-3p and miR-376c-3p were highly expressed, activating the Wnt/β-catenin pathway via targeting SOX6, and promoting malignant progression.

Furthermore, it was found that miR-206 could downregulate the expression of CREB5 and inhibit activation of the PI3K/AKT signalling pathway, thereby preventing HCC growth and metastasis [153]. Another downregulated miR in HCC tissue was miR-34a. It was found to directly interact with and regulate the expression of HDAC, thereby inducing apoptosis in HCC cells [154]. MiR-148a targeted death receptor-5 (DR-5), which led to downregulation of EMT and PI3K/AKT signalling pathways and inhibition of HCC cells growth [155]. A recent study demonstrated that miR-222 decreases let-7c expression in HCC cell lines and that this decrease was related to the promotion of retrotransposition of the long interspersed element 1 (LINE-1), thereby contributing to carcinogenesis [156].

Numerous studies have reported marked dysregulation of lncRNAs in HCC. However, the potential roles of these deregulated lncRNAs in disease initiation and progression, as well as the underlying mechanisms, are poorly understood. Below, we present some of the lncRNAs that have been recently investigated in HCV-associated HCC. LncRNA LINC01189 is downregulated in HCV-infected HCC tumours and cell lines, and it acts as a sponge for miR-155-5p, an oncomiR that is involved in the activation of oncogenic signalling pathways such as RAS/MAPK pathway [157]. LncRNA FIRRE promotes hepatocellular carcinoma by HuR-CyclinD1 axis signalling. Its overexpression in hepatocytes induced cell proliferation, colony formation, and xenograft tumour formation [158]. Sur et al. [159] revealed that HCV infection upregulates miR-373 and Wee1, a key regulator of the G2 cell cycle checkpoint. The results showed that miR-373 may interact with the lncRNA NORAD in HCV-infected cells, forming a complex that sequesters miR-373 and relieves repression of their shared target, Wee1, in HCV-infected cells. This promoted uncontrolled cell proliferation. LncRNA CASC11 enhances the stability of one of the central regulators of cell cycle progression, E2F1 mRNA, by recruiting EIF4A3, which leads to the upregulation of E2F1 in HCC cells. This consequently activates the NF-κB signalling and PI3K/AKT pathway, thus promoting HCC cell proliferation and progression [160]. LncRNA CASC2 was found to be downregulated and function as a tumour suppressor in HCC. It was revealed that CAS2 acts as a sponge, inhibiting the expression of miR-155 and consequently upregulating SOCS1 expression, which suppresses cell proliferation, migration, and invasion [161]. In a study on hepatitis B virus (HBV)- and HCV-related HCC, the most downregulated miRs in HCC compared to normal were miR-424-5p, miR-136-3p, miR-139-5p, miR-223-3p, and miR-375-3p [162]. LncRNA-KCNQ1OT1 was found to physically interact and sponge all these miRs, modulating the BMP signalling pathway to promote chemoresistance in HCC. Furthermore, suppression of KCNQ1OT1 by CRISPR technology resulted in regression of oncogenic properties with enhanced chemosensitivity and reduced metastasis in Huh7/SNU449 cells. These results suggest that LncRNA-KCNQ1OT1 might serve as a therapeutic target in HCC. A recent genome-wide association study in Japan identified lncRNA Prader–Willi non-protein-coding RNA 4 (PWRN4) to be associated with post-SVR hepatocellular carcinoma. The AA genotype of rs4778350 enhanced PWRN4 expression, promoting cell proliferation, migration, and invasion [163]. However, data on ncRNAs in HCC arising after SVR remain limited.

CircRNAs exhibit organ-, tissue- and cell-specific expression patterns and are particularly abundant in the liver. In the context of HCC, including HCV-HCC, circRNAs contribute to regulatory networks that control cell proliferation, apoptosis, and tumour progression [164]. In a recent study, Tang et al. [165] found that circRNA-mTOR was overexpressed in HCC and correlated with patient prognosis. The results suggested that circRNA-mTOR was involved, via the PSIP1/c-Myc signalling pathway, in promoting HCC progression and in resistance to lenvatinib. Further investigation revealed that circRNA-mTOR promoted the nuclear translocation of the RNA-binding protein (RBP) PC4 and SRSF1 interacting protein 1 (PSIP1). Another circRNA with tumour-promoting effects in HCC is CircPIAS1. It was found that its overexpression inhibited ferroptosis via the miR-455-3p/NUPR1/FTH1 axis [166]. Through in vitro and in vivo experiments, circLARP1B is identified to play critical roles in HCC. It was revealed that it promotes HCC metastasis and lipid accumulation via the HNRNPD-LKB1-AMPK pathway [167]. Another study providing in vivo and in vitro data showed that circACVR2A promoted the proliferation, migration and invasion of HCC. It was found that circACVR2A can directly interact with miR511-5p and regulate the expression of related proteins in the PI3K-Akt signalling pathway [168]. According to a study by Gu et al. [169], CircIPP2A2 promotes malignant behaviours in HCC by acting as a molecular scaffold that modulates the Hornerin/PI3K/AKT/GSK3β axis. The study demonstrated that CircIPP2A2 is regulated by N7-methylguanosine modification. The same research group also demonstrated that CircITCH, found to be downregulated in HCC cells, may have tumour-suppressive activity by sponging miR-184, resulting in inhibition of HCC progression [170].

Overall, ncRNAs have emerged as important regulators in HCV-associated hepatocarcinogenesis, contributing to viral persistence and the modulation of pathways involved in tumour initiation and progression. However, the extent of their contributions remains incompletely understood due to limited mechanistic insight, as most available data are based on expression profiling rather than functional validation. Moreover, there is a notable lack of studies examining ncRNA profiles and their roles in HCV-related HCC development following SVR, highlighting an important area for future research.

### 3.4. HCC-Specific Epigenetic Alterations and Shared Molecular Mechanisms Across Different Aetiologies

HCV-driven hepatocarcinogenesis arises through chronic cycles of liver injury and regeneration, during which epigenetic alterations contribute to both fibrogenesis and malignant transformation, thereby creating a shared molecular landscape [10,70,89]. This overlap complicates the identification of HCC-specific biomarkers, particularly because methylation changes progressively accumulate during disease progression.

Nevertheless, the previously discussed studies suggest that DNA methylation events associated with CHC progression can be distinguished from those specifically linked to hepatocarcinogenesis. For example, *p16* and *RASSF1A* methylation occur early in CHC and serve as markers of chronic liver injury and field cancerization [74,105,106,107], while hypermethylation of *GSTP1*, *RUNX3*, and *SOCS-1* appears to be tumour-specific and may therefore represent a reliable hallmark of HCC [100,103]. Importantly, a recent large-scale methylation profiling study demonstrated that the DNA methylation landscape of HCV-associated HCC is strongly influenced by the extent of underlying liver damage, with distinct methylation patterns becoming more pronounced in cirrhotic compared with fibrotic liver tissue [113]. However, the precise distinction between fibrosis/cirrhosis-associated and HCC-specific methylation changes remains to be fully elucidated.

Current evidence suggests that chronic liver injury is largely driven by inflammation-associated alterations, while malignant transformation involves more global and persistent chromatin remodelling. Accordingly, Hamdane et al. [75] demonstrated that the histone modification H3K27ac functions as a mediator of malignant transformation rather than merely a bystander effect of cirrhosis, reflecting persistent structural remodelling of the liver. Importantly, these epigenetic alterations persist after DAA-mediated viral clearance, particularly in advanced fibrosis and cirrhosis, and independently predict HCC development irrespective of fibrosis stage. Moreover, H3K27ac-regulated genes, including *SOX9* and *SPHK1*, promote tumour proliferation and progression in experimental models, supporting the potential role of H3K27ac as a functional biomarker of HCC.

Although the distinction is not clear-cut, current evidence suggests that certain ncRNAs, such as miR-122 and miR-155, are primarily associated with CHC-related inflammation and fibrogenesis, while others, including miR-21, miR-221/222, and miR-92a, become more prominently dysregulated during malignant transformation and HCC progression [136,137,138].

Overall, differentiating HCC-specific epigenetic alterations from background liver disease-associated changes is crucial for the development of clinically relevant biomarkers and the translation of epigenetic findings into effective HCC risk assessment and early detection strategies.

While HCC surveillance traditionally targets cirrhotic patients, non-cirrhotic individuals with HCV infection, particularly those with advanced fibrosis, also require consistent monitoring. In this population, HCC risk is further increased by metabolic comorbidities such as diabetes mellitus and hepatic steatosis [171]. These findings highlight the importance of integrating clinical and metabolic risk factors into individualised HCC surveillance strategies.

In recent years, alongside chronic HCV infection, metabolic dysfunction-associated steatotic liver disease (MASLD) and metabolic dysfunction-associated steatohepatitis (MASH) have emerged as major contributors to HCC development [171]. Importantly, many of the epigenetic alterations described in HCV-related hepatocarcinogenesis also appear to be shared across MASLD/MASH-associated HCC, reflecting common molecular responses to chronic inflammation and metabolic dysregulation [76,172]. Although certain epigenetic alterations may be aetiology-specific, accumulating evidence suggests substantial overlap in the molecular pathways driving hepatocarcinogenesis across chronic liver diseases. Moreover, evidence indicates that HCC arising from these aetiologies involves overlapping dysregulated signalling pathways [173,174]. Therefore, comparing HCV-induced epigenetic alterations with those observed in MASLD/MASH-associated HCC may help identify common epigenetic mechanisms underlying hepatocarcinogenesis across different aetiologies.

Similar to the HCV-induced epigenetic changes that persist after SVR, recent evidence suggests that metabolically driven epigenetic signatures in MASLD patients may persist even after improvement in metabolic status, reflecting a phenomenon referred to as metabolic memory [175,176].

This epigenetic reprogramming may establish a persistent premalignant state that remains independent of the removal of the underlying aetiological trigger. Furthermore, Jühling et al. [76] demonstrated that CHC and non-alcoholic steatohepatitis (NASH) (recently renamed to MASH) share similar epigenetic and transcriptomic alterations associated with liver disease progression and increased HCC risk. Specifically, both aetiologies share a common set of enhancers and H3K27ac modifications that drive the expression of key oncogenes.

Collectively, these findings suggest that hepatocarcinogenesis may rely on partially conserved epigenetic mechanisms across different chronic liver diseases, supporting the development of future epigenetic-based strategies for HCC prevention, risk stratification, and early detection. Nevertheless, the extent to which these epigenetic signatures are universally shared or remain aetiology-specific has yet to be fully elucidated, highlighting the need for further longitudinal and mechanistic studies to improve their translational applicability.

## 4. Epigenetic Mechanisms of HCC Post-HCV Clearance

The introduction of DAA therapy significantly improved HCV outcomes, with cure rates now exceeding 95% [177]. Nevertheless, achieving SVR does not completely eliminate the risk of HCC. This risk is similar to what was noticed with older IFN-based therapies [178,179,180,181,182,183,184,185]. Consequently, the occurrence of HCC following SVR represents a growing clinical challenge, prompting extensive research focused on early detection and preventive strategies [89].

Several studies have observed that patients with HCV-related cirrhosis are at increased risk of developing HCC after DAA treatment. However, HCC may also arise in individuals with mild-to-moderate liver fibrosis [113,186,187,188].

The processes underlying hepatocarcinogenesis after DAA therapy remain unclear. However, potential mechanisms include advanced liver injury, epigenetic scars, and other host-specific factors [189,190]. While other oncogenic pathways are summarised elsewhere [10,89,186], this review focuses specifically on HCV-induced epigenetic mechanisms that contribute to post-SVR HCC risk.

Growing evidence suggests that HCV-induced epigenetic modifications, often termed an epigenetic signature, can persist in the host genome even after viral clearance. Comprehensive genome-wide analyses across multiple models have established that HCV triggers permanent epigenetic reprogramming in the host genome by altering histone modification patterns. Such modifications cause stable changes in gene expression that persist even after successful DAAs or IFN-based therapy, potentially driving the sustained risk of HCC development in cured patients.

Perez et al. [71] reported that HCV induces extensive alterations in the chromatin markers H3K9ac, H3K9me3, and H3K4me3, resulting in reprogrammed host gene expression and altered signalling pathways implicated in hepatocarcinogenesis. These modifications persist as an epigenetic signature even after viral eradication by DAA treatment, as confirmed in both cell culture and clinical biopsies. The authors proposed an eight-gene signature that is highly correlated with enrichment of H3K9ac and H3K4me3. Among these, *WNT10A*, *JUNB*, *FOSL2*, *MYCN*, *TNFAIP3*, *KLF4* and *EDN1* are upregulated, while *PCSK9* is downregulated. They further demonstrated that H3K9ac enrichment could be reversed in vitro by C646, a p300/CBP inhibitor, and by erlotinib, an epidermal growth factor receptor (EGFR) inhibitor, as HCV activates the EGFR signalling pathway.

In a broader study, Perez and colleagues [128] proposed a twelve-gene signature, notably featuring the proliferation regulators *JAK2* and *MTMR9*, and the DNA repair regulator *ATM*. It was demonstrated that there was a high frequency of C-to-T transversions at these gene loci in HCV-associated HCC cases, probably the consequence of HCV-induced reduction in the expression of enzymes involved in base excision repair. These genes were highly correlated with the H3K4me3, H3K9ac, or H3K9me3 biomarkers. H3K4me3 and H3K9ac, markers of open chromatin, were increased, whereas H3K9me3, a marker of closed chromatin, was decreased.

In parallel, other studies have observed that HCV infection induces persistent H3K27ac repositioning across the genome, which remains even after viral clearance with DAAs or IFN-based therapies. These epigenetic changes, which affect key cancer-related genes at both the mRNA and protein expression levels, have been validated across cell culture models, human liver chimeric mouse models and clinical biopsies [71,75]. In their study, Juhling et al. [76] demonstrated that HCV-induced H3K27ac enrichment increased expression of tumourigenesis regulators *FGFR1*, *CCND2*, *MLLT3*, *CDH11*, and *MAML2*. On the other hand, when H3K27ac decreased, the expression of tumour-suppressor genes *FANCC* and *TSC2* also decreased. The authors also demonstrated that JQ1 slowed HCC progression in mouse models. JQ1 is an inhibitor of bromodomain 4 (BRD4), a regulator of transcription elongation that blocks the binding of BRD4 to H3K27ac. In addition, Hamdane et al. [75] observed epigenetic alterations in the adjacent non-tumourous liver tissue of HCC patients, suggesting these are an early step in tumour formation. Overall, the results to date reveal that HCV-induced histone modifications remodel chromatin, influencing the expression of genes that regulate cell proliferation, apoptosis, angiogenesis, immune response and cell repair mechanisms. The observed high-HCC risk signatures require validation on a larger number of samples. The main challenge with these alterations is that they persist as epigenetic scars after successful antiviral treatment. This sheds light on the importance of surveillance of CHC patients after DAA therapy, especially those with advanced stages of liver damage, such as fibrosis and cirrhosis.

Sugimachi et al. [77] conducted genome-wide methylome and transcriptome profiling of tissue samples from patients with SVR-HCC. Their findings revealed that HCV induces persistent methylation alterations that affect transcription factors, subsequently altering the expression of target genes involved in apoptosis and inflammation in both active HCV and post-SVR HCC cases, suggesting their role in driving carcinogenesis after HCV eradication. Research by Hlady et al. [127] using cell line models demonstrated that HCV triggers epigenetic remodelling, characterised by simultaneous changes in DNA methylation and histone modifications, particularly within enhancer regions. Concurrently, HCV induces host immune suppression by epigenetically silencing the *TLR3* gene, thereby reducing hepatocyte sensitivity to apoptotic signals. Furthermore, Oltmanns et al. [191] reported that CHC infection accelerates epigenetic ageing, reflected in the altered methylation status at specific CpG sites. The authors observed that this state was not reversed in patients who developed HCC after DAA-induced SVR, and suggested that this process contributes to HCC development.

## 5. Clinical Significance of Epigenetic Biomarkers in the Monitoring of Post-SVR Patients

Because the risk of HCC persists even after SVR, identifying reliable molecular markers is essential for early cancer prediction and prevention. Although various risk-stratification models have been developed by integrating clinical, molecular, and radiological data, these tools remain highly heterogeneous [192,193,194,195,196]. Consequently, there is a critical need for more rigorous validation and further research to establish precise predictive models for clinical use. Given that current serum biomarkers lack sufficient specificity, as reviewed in the recent study by Yang et al. [197], epigenetic alterations are increasingly being investigated as alternative independent or supplementary markers.

### 5.1. Epigenetic Biomarkers in Early HCC Detection and Prevention

As mentioned previously, numerous studies have shown that aberrant promoter methylation of specific genes can be detected in plasma or serum DNA, as well as in tissue DNA, of HCV-related liver conditions. This provides an opportunity to design non-invasive blood tests for the detection of methylation markers and to identify post-SVR patients who will eventually develop HCC [74,101,102,107,198].

Recently, Sugimachi et al. [77] proposed a set of candidate genes, including those encoding transcription factors (*RXRA*, *KLF4*, *RUNX1*, and *RORA*) and their respective targets (*HSD17B2*, *CXCL12*, *TF*, *TNFRSF10B*, *TRIB3*, *CDH5*, *IFITM3*, *SOD1*, *ATF3*, and *TUBA1B*). These genes exhibit aberrant methylation patterns in post-SVR HCC patients and show potential as non-invasive serum biomarkers for the prediction and early detection of cancer in HCV-cured patients. In another study, Oltmanns et al. [191] suggested that “epigenetic ageing”, a measure of changes in DNA methylation patterns that estimates how fast cells and tissues are ageing, may serve as a biomarker for HCC risk after HCV eradication. Specifically, the authors examined genome-wide DNA methylation status in peripheral blood mononuclear cells (PBMCs) and found that patients who later develop HCC exhibit persistent epigenetic age acceleration. Nishikawa et al. [199] proposed that upregulation of the *TMEM164* gene, driven by promoter DNA demethylation, is a potential diagnostic biomarker for HCC development in post-SVR patients.

In addition to serum and plasma, PBMCs provide a reliable alternative for tumour-related biomarker discovery. By combining PBMC gene expression profiling with in silico analyses, Moustafa et al. [200] identified a promising non-invasive panel consisting of *JUNB*, *WNT10A*, *SPHK1*, *EDN1*, and *KLF4* genes in DAA-SVR patients, predominantly dysregulated by histone modifications. The use of these biomarkers could enhance early detection, prognosis, and personalised therapy compared with conventional markers such as alpha-fetoprotein (AFP).

Several studies have reported that HCV triggers specific genome-wide alterations in key histone markers (H3K9Ac, H3K4Me3 and H3K9Me3) [71,75,76]. As these modifications affect transcription of target genes and persist even after successful DAA treatment, they can serve as predictive biomarkers to identify patients at higher risk of developing HCC. Specifically, Perez et al. [71] proposed an eight-gene signature model consisting of *WNT10A*, *JUNB*, *FOSL2*, *MYCN*, *TNFAIP3*, *KLF4*, *EDN1*, and *PCSK9* to evaluate the risk of HCC development in patients before and after achieving SVR. Through integrative analyses of CHC patients and experimental models, Juhling et al. [76] proposed a twenty-five-gene signature that predicts high HCC risk after DAA treatment. The authors also identified chromatin readers as promising chemopreventive targets, demonstrating their potential to reverse the aberrant gene expression and thus reduce the risk of cancer development.

Additionally, a wide range of ncRNAs analysed in hepatic tissue, serum/plasma or extracellular vesicles have been proposed as potential biomarkers for HCV-related hepatocarcinogenesis [140,201,202,203,204]. A recent study by Elgedawy et al. [205] proposed circulating miR-485-5p as a biomarker for early detection and prediction of prognosis in patients with HCV-HCC. Moreover, panels consisting of several miRs solely or in combination with conventional HCC markers such as AFP are frequently proposed [206]. In addition, lncRNAs and circRNAs are also considered to have significant potential as biomarkers for HCV-HCC. Some recent examples are lncRNAs LINC01564 and RAMS11, MALAT1 and HOTTIP, NBAT-1, and FOXCUT [207,208,209]. Due to their stability and presence in body fluids, circRNAs are increasingly being explored as potential biomarkers for diagnosis and prognosis. A panel consisting of hsa_circ_0003288, circ-RNF13, circANRIL, circUHRF1, and hsa_circ_103047 was proposed as a diagnostic biomarker and therapeutic target for HCV-induced HCC [210].

In line with accumulating preclinical evidence, a substantial number of clinical trials are exploring ncRNAs as biomarkers for diagnosis, prognosis, and treatment response in HCC [140]. Moreover, a number of studies have identified distinct microRNA expression profiles between treatment-naive HCC and HCC arising after DAA therapy, highlighting their potential as non-invasive biomarkers specifically for HCC following HCV eradication. There is evidence that specific ncRNAs, such as lnc-HOTAIR, and various miRNAs, including miR-3197, miR-4718, miR-642a-5p, miR-6826-3, and miR-762, represent potential biomarkers for predicting HCC occurrence following DAA therapy [211,212,213]. Damjanovska et al. [214] performed small RNASeq profiling of plasma samples from DAA-treated patients who developed HCC and from those who remained HCC-free in the 3 years following treatment. The profiling was conducted at several time points during this period, including baseline (before therapy started) and 12 weeks after therapy completion. Different small RNA profiles were found between the two groups and between various time points within each group. Among them, miR-576, miR-664B, miR-7848, and miR-1292 showed a very good ability to discriminate the HCC and control samples throughout the time points used in this study. Furthermore, Pascut et al. [213] suggested that miR-3197 may serve as a potential serum biomarker for identifying high-risk individuals following DAA therapy, especially among those with HCV-induced liver cirrhosis. Me et al. [215] conducted a study involving 950 patients with HCV-related chronic liver disease who underwent DAA treatment. Serum miRNA-135a in combination with IL-13 and vitamin D were identified as potential biomarkers in monitoring DAA treatment and HCC prediction. Another study from Egypt identified serum miR-21-5p, miR-122-5p, and miR-222-3p as biomarker candidates for HCC post-DAA therapy [216]. In a study analysing serum levels of the let-7 family in patients with CHC who developed HCC after antiviral therapy, circulating let-7i emerged as a promising biomarker for early surveillance in patients with HCC risk after antiviral treatment [217]. More recently, Teng et al. [218] have assessed serum extracellular vesicle miRNA profiles (EV-miRNAs) associated with HCC development following SVR. They identified baseline serum EV-miR-1-3p as a protective biomarker for stratifying HCC risk and predicting survival in CHC patients after HCV eradication via DAA therapy, in addition to discovering that patients with high EV-miR-1-3p levels showed higher platelet counts and albumin, and a lower proportion with FIB-4 ≥ 3.25, suggesting that high EV-miR-1-3p reflects better preserved liver function and less advanced fibrosis.

Although ncRNAs show considerable promise as biomarkers for the diagnosis and prognosis of HCC, including post-SVR HCC, their clinical utility still requires validation through large-scale, multicentre case–control studies.

### 5.2. Epigenetic Biomarkers as Targets or Therapeutics for HCC Therapy

The reversible nature of epigenetic modifications makes them promising therapeutic targets. Consequently, the use of specific inhibitors that reverse the epigenetic signature could potentially reduce the oncogenic effects of CHC infection [71,219].

Several studies have reported that HCV-induced epigenetic alterations persisting after successful DAA cure can be reversed by epigenetic drugs [71,75,76]. Current approaches to epigenetic therapy for the treatment and prevention of HCC involve several key strategies: DNMT and HDAC inhibitors, histone methyltransferase (HMT) inhibitors, curaxins, a specific class of anticancer agents, and RNA-based therapies. In addition, recent research is increasingly focused on the potential of combined treatments that integrate epigenetic drugs with immunotherapy, as recently reviewed [172].

Initially approved for the treatment of haematological tumours, DNMT and HDAC inhibitors have proven to be potent, multifaceted therapeutic agents. Results to date have shown that these epidrugs can reprogram cancer stemness through multiple pathways and are effective at low, less-toxic doses compared with chemotherapy. In addition, they can restore the sensitivity of malignant cells to standard treatments. By targeting the tumour microenvironment, these epidrugs also enhance immune recognition and synergise effectively with immunotherapy (reviewed in [219]). However, despite these promising effects, in the context of HCV-induced hepatocarcinogenesis, epigenetic drugs often lack specificity, exerting genome-wide effects rather than precisely targeting the modifications induced by the virus [71]. Therefore, ongoing research aims to identify the mechanisms mediating HCV interaction with the host epigenetic machinery, known as epigenetic modifiers, and to explore their therapeutic potential. Encouraging results have already been observed regarding the EGFR signalling pathway, which plays a critical role in HCV-related liver pathogenesis [220]. Research indicates that HCV specifically activates this pathway, leading to histone acetylation and a persistent epigenetic signature in DAA-treated cells, which can be effectively reversed by erlotinib, an EGFR inhibitor, thereby reducing its oncogenic potential [71]. Another possible mechanism involves targeting the HCV-induced unfolded protein response (UPR), which drives epigenetic alterations associated with an increased risk of HCC development. Using UPR-specific inhibitors has been shown to partially reduce the expression of cancer-related genes [76]. Although these epigenetic modifiers offer a promising strategy for HCC prevention, further research is required to confirm their effectiveness in clinical practice [89].

NcRNA-based therapeutic strategies offer great potential in the treatment of cancer because they operate at a fundamental regulatory level of gene expression, which enables precise modulation of entire oncogenic networks instead of targeting individual proteins. These molecules can simultaneously influence multiple genes and signalling pathways that are implicated in tumour initiation, progression, and metastasis. Additionally, ncRNA therapies can be engineered to restore tumour-suppressive functions or inhibit oncogenic drivers that are frequently considered “undruggable” by conventional small molecules or antibodies [221]. Moreover, because ncRNAs broadly reprogram cellular networks, these therapies might prevent or delay the emergence of drug resistance, which often accompanies conventional treatments [221].

MiRNAs are the most extensively studied as potential therapeutic agents, owing to their well-characterised roles in post-transcriptional gene regulation and their ability to simultaneously modulate multiple targets within key oncogenic pathways [146,149,221,222]. Therapeutic strategies involving miRNAs have focused on either restoring tumour-suppressive miRNAs or inhibiting oncogenic miRNAs to suppress cancer progression. The first miRNA that was ever tested in a therapeutic context in a clinical trial involving cancer patients was miR-34a. Acting as a tumour-suppressor microRNA that controls cell-cycle arrest, apoptosis, and DNA damage responses, it is often downregulated in various cancers [146]. A phase I human trial (NCT01829971) on multiple cancer types including HCC assessed a liposomal miR-34a mimic—MRX34. Although it demonstrated target engagement and dose-dependent modulation of miR-34a target genes in patients’ white blood cells, the study was terminated due to severe immune-related toxicities, highlighting both the potential and risks of miRNA-based therapies [223].

Other tumour-suppressor miRNAs, such as miR-199a-3p, miR-22, and miR-26a have shown significant therapeutic potential in preclinical studies of HCC [224,225,226]. On the other hand, there is a number of oncomiRs that are being evaluated as therapeutic targets, including miR-21, miR-221/222, miR-155, miR-17-92, and miR-224 [221]. Interestingly, a simultaneous inhibition of an oncomiR and restoration of a tumour-suppressor miRNA, achieved through co-delivery of an anti-miR-21 and a miR-122 mimic to a HCC mouse model, resulted in stronger tumour suppression compared to either treatment alone, highlighting the potential of multi-miRNA modulation to more effectively reprogram tumour-associated regulatory networks [227]. In addition, strategies combining miRNA therapeutics and conventional treatments are another promising field of exploration [228].

Although preclinical findings are promising, the clinical translation of miRNA-based therapies for HCC is still in its early stages [146,221]. Key challenges include inefficient and non-specific delivery to target tissues, poor stability, limited intracellular release and functional activity, and the risk of off-target effects [149]. Additional concerns involve immune activation and toxicity, as seen in the MRX34 trial mentioned above. Nevertheless, recent advances in delivery technologies, particularly liver-targeted systems such as lipid nanoparticles and engineered exosomes, have improved the stability, specificity, and overall clinical applicability of ncRNA-based therapies [229,230]. Furthermore, a recent study demonstrated that full chemical modification of the miR-34a duplex enhanced its stability, target gene repression, and antitumour efficacy compared to partially modified counterparts. In addition, conjugation to folate enabled targeted in vivo delivery resulting in sustained silencing of oncogenic targets and improved tumour suppression at low doses without inducing significant immune activation [231]. These findings highlight the potential of chemically optimised, ligand-directed miRNA therapeutics to overcome key barriers in clinical translation. With continued advances, miRNA-based therapies could become in the future an integral part of the HCC treatment landscape, used in combination with surgery, kinase inhibitors, and immunotherapies to improve patient outcomes.

## 6. Conclusions

Notwithstanding improvements in antiviral therapy and a better understanding of HCV-driven carcinogenic mechanisms, HCV-related HCC remains one of the major global health issues. Although viral clearance is now achievable, the risk of cancer development persists due to complex interactions between viral and host factors. This review emphasises that persistent genetic and epigenetic alterations, together with HCV-induced immunological imprints, create a molecular environment that favours post-SVR hepatocarcinogenesis. Understanding these ongoing biological changes is crucial for improving post-SVR patient management and for developing novel preventive strategies. Accordingly, future research should focus on identifying robust biomarkers and molecular targets to refine risk stratification in individuals with long-term HCC risk associated with HCV infection.

## Figures and Tables

**Table 1:** Histone modifications in HCV-induced HCC.

Modification	Histone Modification	Chromatin State	Action in HCV Scar	Functional Outcome	References
Acetylation	H3K27ac	Open	Enrichment at promoters and enhancers	↑ *SPHK1* → anti-apoptosis, angiogenesis	[75]
				↑ IFNα	[127]
Acetylation	H3K27ac	Open	Depletion (loss at promoters)	*TLR3* silencing → anti-apoptosis, immune response suppression	[127]
Acetylation	H3K9ac	Open	Enrichment at regulatory elements	Proliferation, angiogenesis, inflammation	[71,128]
Acetylation	H3K9ac	Open	Depletion at exons, introns, and TSSs	Decreased expression of base excision repair pathway genes	[128]
Methylation	H3K4me1	Open	Enrichment at enhancers	↑ IFNα	[127]
Methylation	H3K4me3	Open	Enrichment at exons, introns and promoters	Proliferation, angiogenesis, inflammation	[71,127]
Methylation	H3K9me3	Closed	Loss at promoters and TSSs	Decreased expression of base excision repair proteins	[128]
Methylation	H3K27me3	Closed	Enrichment across the *TLR3* gene locus	*TLR3* silencing → anti-apoptosis, immune response suppression	[127]
Ubiquitination	H2AK119ub	Closed	Loss at the *HOX* promoter	HOX ↑ → proliferation, angiogenesis, inflammation	[129]
Methylation/ phosphorylation	H4R3me2	Open	Loss → blockade of H2AX phosphorylation to γ-H2AX	Double-strand breaks → genomic instability	[130]

Abbreviations: IFNα: Interferon alpha, TLR3: Toll-like receptor 3, H3K27ac: Histone 3 lysine 27 acetylation, H3K9ac: Histone 3 lysine 9 acetylation, H3K4me1: Histone 3 lysine 4 monomethylation, H3K4me3: Histone 3 lysine 4 trimethylation, H3K9me3: Histone 3 lysine 9 trimethylation, H3K27me3: Histone 3 lysine 27 trimethylation, H4R3me2: Histone 4 arginine 3 dimethylation, γ-H2AX: Phosphorylated histone H2AX, ↑: increased gene expression.

## Data Availability

No new data were created or analysed in this study. Data sharing is not applicable to this article.

## Abbreviations

The following abbreviations are used in this manuscript:

CHC	Chronic hepatitis C
circRNA	Circular RNA
DAAs	Direct-acting antivirals
DNMTs	DNA methyltransferases
EMT	Epithelial-to-mesenchymal transition
ER	Endoplasmatic reticulum
HATs	Histone acetyltransferases
HCC	Hepatocellular carcinoma
HCV	Hepatitis C virus
HDAC1	Histone deacetylase 1
HMT	Histone methyltransferase
IFN	Interferon
IRES	Internal ribosome entry site
ISGs	Interferon-stimulated genes
ITAFs	IRES trans-acting factors
LVPs	Lipo-viro particles
lncRNA	Long noncoding RNA
MASLD	Metabolic dysfunction-associated steatotic liver disease
MASH	Metabolic dysfunction-associated steatohepatitis
miR, miRNA	Micro-RNA
NASH	Non-alcoholic steatohepatitis
ncRNAs	Non-coding RNAs
NR	Non-responders
PBLs	Peripheral blood lymphocytes
PBMCs	Peripheral blood mononuclear cells
PEG-IFN/RBV	Pegylated interferon and ribavirin
(RASs)	Resistance-associated substitutions
SVR	Sustained virologic response
UPR	Unfolded protein response
VLP	Viral-like particles
ER	Endoplasmic reticulum
